# ﻿The complete mitochondrial genome of *Aeschrocoristuberculatus* and *A.ceylonicus* (Hemiptera, Pentatomidae) and its phylogenetic implications

**DOI:** 10.3897/zookeys.1160.100818

**Published:** 2023-05-09

**Authors:** Wang Jia, Jiufeng Wei, Minmin Niu, Hufang Zhang, Qing Zhao

**Affiliations:** 1 College of Plant Protection, Shanxi Agricultural University, Taigu 030801, Shanxi, China Shanxi Agricultural University Taigu China; 2 Department of Biology, Xinzhou Teachers University, Xinzhou 034000, Shanxi, China Xinzhou Teachers University Xinzhou China

**Keywords:** Mitogenome, Pentatomoidea, phylogenetic analysis

## Abstract

*Aeschrocoristuberculatus* and *A.ceylonicus* (Hemiptera, Pentatomidae, Pentatominae) are mainly distributed in southern China, India, Myanmar, and Sri Lanka. Both species are also common agricultural pests. However, only the morphology of the genus *Aeschrocoris* has previously been studied, and molecular data have been lacking. In this study, the whole mitochondrial genomes of *A.tuberculatus* and *A.ceylonicus* are and annotated. The lengths of the complete mitochondrial genomes of the two species are 16,134 bp and 16,142 bp, respectively, and both contain 37 typical genes, including 13 protein-coding genes (PCGs), two ribosomal RNA genes (rRNAs), 22 transfer RNA genes (tRNAs), and a control region. The mitochondrial genome structure, gene order, nucleotide composition, and codon usage of *A.tuberculatus* and *A.ceylonicus* are consistent with those of typical Pentatomidae. Most PCGs of both species use ATN as the start codon, except *atp8*, *nad1*, and *cox1*, which use TTG as the start codon. *cox1*, *cox2*, and *atp6* use a single T, and *nad1* use TAG as the stop codon; the remaining PCGs have TAA as the stop codon. The A+T contents of the two species are 73.86% and 74.08%, respectively. All tRNAs have a typical cloverleaf structure, with the exception of *trnS1*, which lacks a dihydrouridine arm. The phylogenetic tree is reconstructed using the maximum-likelihood method based on the newly obtained mitochondrial genome sequences and 87 existing mitochondrial genomes of Pentatomoidea from the NCBI database and two species of Lygaeoidea as outgroups. The phylogenetic trees strongly support the following relationships: (Urostylididae + ((Acanthosomatidae + ((Cydnidae + (Dinidoridae + Tessaratomidae)) + (Scutelleridae + Plataspidae))) + Pentatomidae). This study enriches the mitochondrial genome database of Pentatomoidea and provides a reference for further phylogenetic studies.

## ﻿Introduction

The insect mitochondrial genome is a circular double-stranded DNA molecule with a length of about 16–18 kb, which code 37 genes: 13 protein-coding genes (PCGs), two ribosomal RNA (rRNA) genes, and 22 transfer RNA (tRNA) genes (Boore 1999). In addition, the mitochondrial genome usually includes a noncoding region of variable length that plays a regulatory role in transcription and replication, known as the mitochondrial control region (Cameron 2014). In recent years, with the development of sequencing technology and the amplification through universal primers for mitochondrial genes (Simon et al. 1994, 2006), the number of insect mitochondrial genomes has rapidly increased, and the characteristics and evolutionary patterns of insect mitochondrial genomes are becoming more and more clear; their applications in phylogenetic studies are gradually increasing. The mitochondrial genome contains important molecular evolutionary information such as base composition and codon usage (Yuan et al. 2022). It has been widely used in research on molecular evolution, phylogeny, genealogy, and population genetic structure because of its stable gene composition, relatively conserved order, matrilineal inheritance, and minimal recombination (Ballard and Whitlock 2004; Simon and Hadrys 2013; Cameron 2014).

Pentatomoidea (Hemiptera, Heteroptera, Pentatomomorpha) consists of more than 8,000 species in 18 families, of which Pentatomidae is the largest family containing 940 genera and about 5,000 species (Rider et al. 2018). Pentatomidae is a species-rich group, so it is difficult to propose defining characteristics that can be applied to all groups. All stinkbugs of Pentatomidae are terrestrial insects, most of which are phytophagous; only Asopinae are predatory species, and some are used as biological control agents (De Clercq et al. 2003).

The tribe Aeschrocorini was first proposed by Distant (1902) and included two genera, *Aeschrocoris* Bergroth, 1887 and *Scylax* Distant, 1887. It remained little known until Cachan (1952) added a new genus to the tribe. The Aeschrocorini is still relatively small, currently with only eight genera (Rider et al. 2018). Hassan et al. (2016) provided a brief record of Indian species of *Aeschrocoris*. Despite the complex taxonomic relationships within the Aeschrocorini, numerous scholars have consistently assigned the genus *Aeschrocoris* to Aeschrocorini (Rider et al. 2018). *Aeschrocoris* was reported to have five species in China and eight in the world. *Aeschrocoristuberculatus* (Stål, 1865) and *A.ceylonicus* Distant, 1899 are mainly distributed in southern China, India, Myanmar, and Sri Lanka, and both are also common agricultural pests (Fan 2011). However, most studies of the genus *Aeschrocoris* have focused on morphological descriptions and lack molecular data.

In this study, we analyze the mitochondrial genomes of *A.tuberculatus* and *A.ceylonicus* in detail, including genome structure, nucleotide composition, and codon usage. Meanwhile, we also construct the genome structure of RNA. In addition, we analyze the phylogenetic relationship of eight families of Pentatomoidea and explore the phylogenetic location of these two species. The results of this study will provide a reference for phylogenetic analyses and identification of the Pentatomoidea.

## ﻿Materials and methods

### ﻿Sample collection

Adult specimens of *Aeschrocoristuberculatus* and *A.ceylonicus* were collected from Baihua Ling (Baoshan City, Yunnan Province, China; 25°16'43"N, 98°48'12"E) on 13 August 2015 and from Guanlan Ting (Taohua Island, Zhoushan City, Zhejiang Province, China; 29°50'31"N, 122°14'13"E) on 4 August 2016. All samples were immediately placed in anhydrous ethanol and stored in a refrigerator at –25 °C until DNA was extracted. The species were identified by Qing Zhao.

### ﻿DNA extraction and sequencing

Whole-genome DNA was extracted from the thoracic muscle of the samples using the Genomic DNA Extraction Kit (BGI, Wuhan, Hubei, China). Concentrations of samples were detected using Qubit Fluorometer and microplate reader (Mardis and McCombie 2017). The integrity of the samples was tested by agarose gel electrophoresis. High-throughput pair-ended sequencing (PE150) was performed on DNBSEQ platform for the complete mitochondrial genomes of the two species (Chen et al. 2018). All the above operations were carried out in the high-throughput laboratory at Wuhan BGI Technology Services Co., Ltd. (Wuhan, Hubei, China).

### ﻿Genome annotation and sequence analysis

When the assembly was complete, the complete mitogenomes were manually annotated using Geneious v. 11.0 software (Kearse et al. 2012). Two reference sequences (*Eurydemagebleri* and *Brachymnatenuis*) for annotation were obtained from the Basic Local Alignment Search tool (BLAST) in the NCBI database. The boundaries of the PCGs were determined using Open Reading Frame Finder on the NCBI website (http://www.ncbi.nlm.nih.gov/gorf/gorf.html). MEGA v. 11.0 (Tamura et al. 2021) was used to translate the proteins to verify the start codons, stop codons, and amino acid sequences and to ensure the accuracy of the sequences. We annotated tRNA sequences using tRNAscan-SE 2.0 (http://lowelab.ucsc.edu/tRNAscan-SE/; Lowe and Eddy 1997) or used automatic annotation done by MITOS (http://mitos.bioinf.uni-leipzig.de/index.py/; Bernt et al. 2013) with the invertebrate mitochondrial code. The boundaries of the rRNA genes were completed based on the positions of adjacent genes and published rRNA gene sequences (Boore 2006). The control region was identified through the boundary of the neighboring genes.

The base composition, codon usage (RSCU), and amino acid composition of the mitogenome were analyzed using MEGA v. 11.0. The skew of the nucleotide composition was calculated as follows: AT-skew = (A – T) / (A + T) and GC-skew = (G – C) / (G + C) (Perna and Kocher 1995; Hassanin et al. 2005; Bernt et al. 2013). DnaSP6 software (Rozas et al. 2017) was used to count the non-synonymous substitutions (Ka) and synonymous substitutions (Ks) of 13 PCGs of Pentatomoidea and to calculate the Ka/Ks values. The ratio Ka/Ks indicated the rate of evolution, the higher the ratio, and the faster the rate of evolution.

### ﻿Phylogenetic analyses

In this study, we used the two newly sequenced species, 87 species from other eight families of Pentatomoidea, and two species (*Geocorispallidipennis* and *Kleidocerysresedae* as the outgroup) from Lygaeoidea to analyze the phylogenetic position of *A.tuberculatus* and *A.ceylonicus* and the phylogenetic relationships within Pentatomoidea (Table 1). DNA alignment was inferred from the amino-acid alignment of the 13 PCGs using MUSCLE with default settings in MEGA v. 11 (Edgar 2004).

**Table 1.** List of species used to construct the phylogenetic tree.

Classification	Family	Species	Accession number	Reference
**Outgroup**				
Lygaeoidea	Lygaeidae	*Geocorispallidipennis*	EU427336	Hua et al. 2008
		*Kleidocerysresedae*	KJ584365	Li et al. 2016
**Ingroup**				
Pentatomoidea	Acanthosomatidae	*Acanthosomalabiduroides*	JQ743670	Li et al. 2017
		*Anaxandrataurina*	NC042801	Liu et al. 2019
		*Sastragalaedessoides*	JQ743676	Li et al. 2017
		*Sastragalaesakii*	MW847247	Xu et al. 2021
	Cydnidae	*Adrisamagna*	NC042429	Liu et al. 2019
		*Aethusnigritus*	MW847231	Xu et al. 2021
		*Macroscytusgibbulus*	EU427338	Hua et al. 2008
		*Macroscytussubaeneus*	MW847241	Xu et al. 2021
		*Scoparipessalvazai*	NC042800	Liu et al. 2019
	Dinidoridae	*Coridiusbrunneus*	MW899158	Unpublished
		*Cyclopeltaparva*	NC037739	Jiang 2017
		*Megymenumgracilicorne*	NC042810	Liu et al. 2019
	Pentatomidae	***Aeschrocorisceylonicus***	OP526368	This study
		***Aeschrocoristuberculatus***	OP526367	This study
		*Armacustos*	NC051562	Wu et al. 2020
		*Anaxilausmusgravei*	NC061538	Unpublished
		*Brachymnatenuis*	NC042802	Liu et al. 2019
		*Carbulasinica*	NC037741	Jiang 2017
		*Catacanthusincarnatus*	NC042804	Liu et al. 2019
		*Caystrusobscurus*	NC042805	Liu et al. 2019
		*Cazirahorvathi*	NC042817	Liu et al. 2019
		*Dalpadacinctipes*	NC058967	Xu et al. 2021
		*Dalsirascabrata*	NC037374	Jiang 2017
		*Deroploaparva*	NC063299	Unpublished
		*Dinorhynchusdybowskyi*	NC037724	Zhao et al. 2018
		*Dolycorisbaccarum*	NC020373	Zhang et al. 2013
		*Eocantheconafurcellata*	MZ440302	Unpublished
		*Eocantheconathomsoni*	NC042816	Liu et al. 2019
		*Eurydemadominulus*	NC044762	Zhao et al. 2019b
Pentatomoidea	Pentatomidae	*Eurydemagebleri*	NC027489	Yuan et al. 2015
		*Eurydemaliturifera*	NC044763	Zhao et al. 2019b
		*Eurydemamaracandica*	NC037042	Zhao et al. 2017b
		*Eurydemaoleracea*	NC044764	Zhao et al. 2019b
		*Eurydemaqinlingensis*	NC044765	Unpublished
		*Eurydemaventralis*	MG584837	Unpublished
		*Erthesinafullo*	NC042202	Ji et al. 2019
		*Eysarcorisaeneus*	MK841489	Zhao et al. 2019a
		*Eysarcorisannamita*	MW852483	Li et al. 2021
		*Eysarcorisgibbosus*	MW846868	Li et al. 2021
		*Eysarcorisguttigerus*	NC047222	Chen et al. 2020
		*Eysarcorismontivagus*	MW846867	Li et al. 2021
		*Eysarcorisrosaceus*	MT165687	Li et al. 2021
		*Glauciasdorsalis*	NC058968	Xu et al. 2021
		*Gonopsisaffinis*	NC036745	Chen et al. 2017
		*Graphosomarubrolineatum*	NC033875	Wang et al. 2017
		*Halyomorphahalys*	NC013272	Lee et al. 2009
		*Hippotiscusdorsalis*	NC058969	Xu et al. 2021
		*Hoplistoderaincisa*	NC042799	Liu et al. 2019
		*Menidaviolacea*	NC042818	Liu et al. 2019
		*Nezaraviridula*	NC011755	Hua et al. 2008
		*Neojurtinatypica*	NC058971	Xu et al. 2021
		*Palomenaviridissima*	NC050166	Unpublished
		*Pentatomametallifera*	NC058972	Xu et al. 2021
		*Pentatomarufipes*	MT861131	Zhao et al. 2021
		*Pentatomasemiannulata*	NC053653	Unpublished
		*Picromerusgriseus*	NC036418	Zhao et al. 2017a
		*Picromeruslewisi*	NC058610	Mu et al. 2022
		*Placosternumurus*	NC042812	Liu et al. 2019
		*Plautiacrossota*	NC057080	Wang et al. 2019
		*Plautiafimbriata*	NC042813	Liu et al. 2019
		*Plautialushanica*	NC058973	Xu et al. 2021
		*Priassusspiniger*	OK546352	Unpublished
		*Scotinopharalurida*	NC042815	Liu et al. 2019
		*Tholosanusproximus*	NC063300	Unpublished
		*Zicronacaerulea*	NC058303	Zhao et al. 2020
	Plataspidae	*Brachyplatyssubaeneus*	MW847232	Xu et al. 2021
		*Calactalugubris*	MW847233	Xu et al. 2021
		*Coptosomabifaria*	EU427334	Hua et al. 2008
		*Coptosomavariegatum*	OP123035	Zhu et al. 2022
		*Megacoptabituminata*	OP123020	Zhu et al. 2022
		*Megacoptacaliginosa*	OP123022	Zhu et al. 2022
		*Megacoptacentronubila*	OP123024	Zhu et al. 2022
		*Megacoptacribraria*	JF288758	Unpublished
		*Megacoptacribriella*	OP123025	Zhu et al. 2022
		*Megacoptadistanti*	OP123028	Zhu et al. 2022
		*Megacoptahorvathi*	OP123029	Zhu et al. 2022
		*Megacoptalobata*	OP123031	Zhu et al. 2022
	Scutelleridae	*Cantaoocellatus*	MF497713	Liu et al. 2019
		*Chrysocorisstollii*	NC051942	Unpublished
		*Eurygastertestudinaria*	NC042808	Liu et al. 2019
		*Poecilocorisdruraei*	MW847246	Xu et al. 2021
	Tessaratomidae	*Dalcanthadilatata*	JQ910981	Li et al. 2017
		*Eusthenescupreus*	NC022449	Song et al. 2013
		*Mattiphussplendidus*	NC053743	Xu et al. 2020
		*Pycanumochraceum*	MW899159	Wang et al. 2021
		*Tessaratomapapillosa*	NC037742	Jiang 2017
	Urostylididae	*Urostylisflavoannulata*	NC037747	Jiang 2017
		*Urolabidahistrionica*	MW847249	Xu et al. 2021
		*Urochelaquadrinotata*	NC020144	Li et al. 2012

To determine whether the sequences contained phylogenetic information, we tested nucleotide substitution saturation and plotted transition and transversion rates against the TN93 distances for two datasets: all codon positions of the 13 PCGs (PCG123) and first and second codon positions of PCGs (PCG12) using DAMBE to further validate the feasibility of constructing a phylogenetic tree (Xia and Xie 2001; Xia and Lemey 2009). Heterogeneity in sequence divergence in the two datasets was analyzed by using AliGROOVE with the default sliding window size (Kück et al. 2014). PartitionFinder was used to provide the best fit model (Kalyaanamoorthy et al. 2017). IQtree v. 1.6.12 was used to construct the ML tree (Nguyen et al. 2015), and node confidence was assessed with 500,000 replications for bootstrap (Hoang et al. 2018). The phylogenetic trees were constructed using two datasets, PCG123 and PCG12. Finally, the generated phylogenetic trees were visualized using the online editing tool Chipolt (https://www.chiplot.online).

## ﻿Results

### ﻿Genomic features

**Figure 1.**
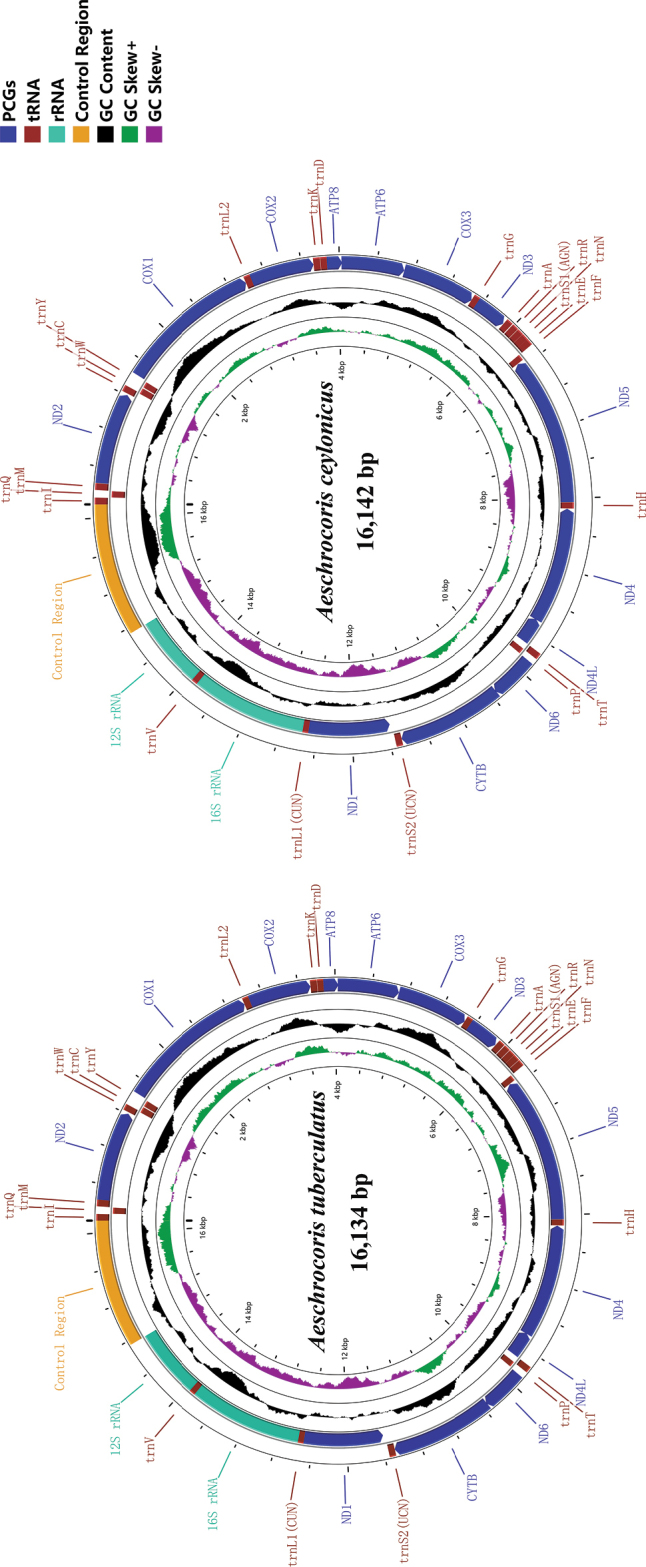
Mitochondrial genome structure of *Aeschrocoristuberculatus* and *A.ceylonicus*. Arrows indicate the orientation of gene transcription. PCGs are shown as blue arrows; tRNAs are named using single-letter amino acid abbreviations.

The complete mitogenomes of *Aeschrocoristuberculatus* (16,134 bp, GenBank accession no. OP56367) and *A.ceylonicus* (14,142 bp, GenBank accession no. OP56368) were obtained (Fig. 1). The mitogenomes of the two species contain a control region and 37 genes (13 PCGs, 22 tRNA genes, and two rRNA genes). The composition of genes is similar to those described in other pentatomid insects (Lee et al. 2009; Zhao et al. 2017a, 2017b; Chen et al. 2019). In addition, the mitochondrial genomes of both species have similar overlapping regions and gene spacer regions. In *A.tuberculatus*, the intergenic overlap region is 34 bp in length and contains seven overlapping regions of 1–8 bp in length. The longest overlapping regions are located between *trnW*/*trnC* and *nad6*/*cytb*. The intergenic spacer is 127 bp in length and contains 17 spacers ranging from 1 to 25 bp in size. The longest spacer (25 bp) is located between *trnS2* and *nad1*. In *A.ceylonicus*, seven intergenic overlapping regions were examined with varying lengths of 1–8 bp, and the longest overlapping region is at the same position (between *trnW* and *trnC*, *nad6*, and *cytb*) as in *A.tuberculatus*. The intergenic spacers are the same in *A.ceylonicus* as in *A.tuberculatus*, and the longest spacer (33 bp) region is also situated between *trnS2* and *nad1* (Table 2).

**Table 2.** Organization of the mitochondrial genomes of *Aeschrocoristuberculatus* and *A.ceylonicus*.

			*A.tuberculatus*					*A.ceylonicus*				
Gene	Strand	Anticodon	Position	Size (bp)	Initiation codon	Stop codon	Intergenic nucleotide	Position	Size (bp)	Initiation codon	Stop codon	Intergenic nucleotide
*trnI*	J	GAT	1–71	71			0	1–71	71			0
*trnQ*	N	TTG	80–148	69			8	79–147	69			7
*trnM*	J	CAT	154–224	71			5	152–222	71			4
*nad2*	J		225–1208	984	ATA	TAA	0	226–1206	981	ATA	TAA	3
*trnW*	J	TCA	1226–1292	67			17	1223–1290	68			16
*trnC*	N	GCA	1285–1352	68			–8	1283–1350	68			–8
*trnY*	N	GTA	1366–1434	69			13	1364–1429	66			13
*cox1*	J		1450–2986	1537	TTG	T	15	1445–2981	1537	TTG	T	15
*trnL2*	J	TAA	2987–3053	67			0	2982–3048	67			0
*cox2*	J		3054–3732	679	ATA	T	0	3049–3727	679	ATA	T	0
*trnK*	J	CTT	3733–3804	72			0	3728–3799	72			0
*trnD*	J	GTC	3808–3873	66			3	3803–3869	67			3
*atp8*	J		3874–4035	162	TTG	TAA	0	3870–4031	162	TTG	TAA	0
*atp6*	J		4029–4701	673	ATG	T	–7	4025–4697	673	ATG	T	–7
*cox3*	J		4702–5490	789	ATG	TAA	0	4698–5486	789	ATG	TAA	0
*trnG*	J	TCC	5490–5555	66			–1	5486–5550	65			–1
*nad3*	J		5556–5909	354	ATT	TAA	0	5551–5904	354	ATT	TAA	0
*trnA*	J	TGC	5914–5982	69			4	5909–5977	69			4
*trnR*	J	TCG	5991–6058	68			8	5988–6056	69			10
*trnN*	J	GTT	6064–6131	68			5	6061–6128	68			4
*trnS1*	J	GCT	6133–6202	70			1	6130–6199	70			1
*trnE*	J	TTC	6207–6275	69			4	6200–6269	70			0
*trnF*	N	GAA	6274–6341	68			–2	6268–6335	68			–2
*nad5*	N		6346–8052	1707	ATG	TAA	4	6340–8046	1707	ATG	TAA	4
*trnH*	N	GTG	8055–8123	69			2	8049–8117	69			2
*nad4*	N		8127–9455	1329	ATG	TAA	3	8121–9449	1329	ATG	TAA	3
*nad4l*	N		9449–9736	288	ATT	TAA	–7	9443–9730	288	ATT	TAA	–7
*trnT*	J	TGT	9739–9807	69			2	9733–9801	69			2
*trnP*	N	TGG	9808–9871	64			0	9802–9865	64			0
*nad6*	J		9880–10353	474	ATA	TAA	8	9868–10347	480	TTG	TAA	2
*cytb*	J		10346–11482	1137	ATG	TAA	–8	10340–11476	1137	ATG	TAA	–8
*trnS2*	J	TGA	11482–11550	69			–1	11476–11534	68			–1
*nad1*	N		11576–12499	924	TTG	TAG	25	11568–12491	924	TTG	TAG	33
*trnL1*	N	TAG	12500–12565	66			0	12492–12557	66			0
*rrnL*	N		12566–13874	1309			0	12558–13859	1302			0
*trnV*	N	TAC	13875–13942	68			0	13860–13927	68			0
*rrnS*	N		13943–14751	809			0	13928–14740	813			0
OH	J		14752–16134	1383			0	14741–16142	1402			0

### ﻿Nucleotide composition and codon usage

The nucleotide composition of two species shows the predominance of A+T in the complete mitochondrial genome (Table 3). The order of base composition of the entire sequence in *A.tuberculatus* and *A.ceylonicus* is A (42.32%) > T (31.55%) > C (15.20%) > G (10.94%) and A (42.36%) > T (31.72%) > C (14.94%) > G (10.98%), respectively. This bias was observed in the complete mitochondrial genome. The A+T content of the two species is 73.09% and 72.79% in PCGs, 76.11% and 76.63% in tRNAs, 75.97% and 76.60% in rRNAs and 73.99% and 77.35% in the control region, respectively. The complete genomes of both also show a clear AC skew (GC skew = –0.16, AT skew = 0.15, GC skew = –0.15, and AT skew = 0.14), suggesting a greater abundance of A than T and a higher abundance of C than G.

**Table 3.** Nucleotide composition of the mitogenomes of *Aeschrocoristuberculatus* and *A.ceylonicus*.

***A.tuberculatus***								
**Feature**	**Length (bp)**	**A**%	**C**%	**G**%	**T**%	**A+T**%	**AT-skew**	**GC-skew**
Whole genome	16134	42.32	15.20	10.94	31.55	73.86	0.15	–0.16
PCGs	11036	32.63	13.54	13.37	40.46	73.09	–0.11	0.01
tRNA	1503	38.39	10.18	13.71	37.72	76.11	0.01	0.15
rRNA	2118	32.39	8.40	15.63	43.58	75.97	–0.15	0.30
Control region	1383	38.41	14.49	11.52	35.58	77.99	0.04	–0.11
***A.ceylonicus***								
**Feature**	**Length (bp)**	**A**%	**C**%	**G**%	**T**%	**A+T**%	**AT-skew**	**GC-skew**
Whole genome	16142	42.36	14.94	10.98	31.72	74.08	0.14	–0.15
PCGs	11040	32.40	13.65	13.56	40.39	72.79	–0.11	0.00
tRNA	1502	38.08	9.85	13.52	38.55	76.63	–0.01	0.16
rRNA	2115	32.77	8.32	15.08	43.83	76.60	–0.14	0.29
Control region	1402	39.40	12.58	10.06	37.96	77.35	0.02	–0.11

**Figure 2.**
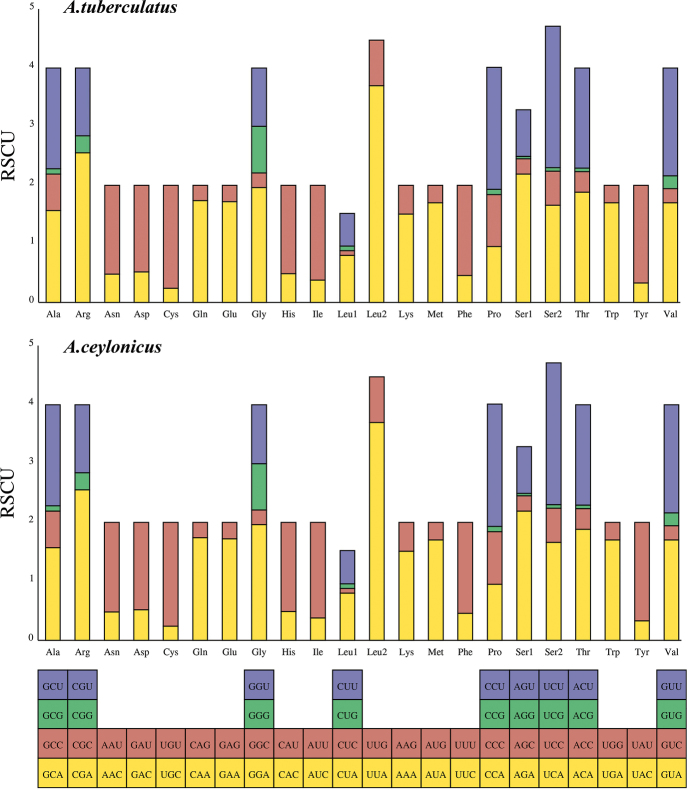
Relative synonymous codon usage (RSCU) within *Aeschrocoristuberculatus* and *A.ceylonicus*. Codon families are shown on the *x*-axis and the frequency of RSCU on the *y*-axis.

The composition of nucleotides is also reflected in the use of codons. The RSCUs of the two species show some differences and are compared to each other in Fig. 2. The most frequently used codons are UUA (Leu2), and most of the codons with high frequency ended in A/T. These results indicate that in the codon composition of *Aeschrocoris* mitogenomes, AT was superior to GC.

### ﻿Protein-coding genes

The length of PCGs in *A.tuberculatus* and *A.ceylonicus* is 11,036 bp and 11,040 bp, respectively. For the 13 PCGs, nine (*cox1*, *cox2*, *cox3*, *atp6*, *atp8*, *nad2*, *nad3*, *nad6*, and *cytb*) are encoded on the major strand (J-strand), whereas the other four are encoded on the minor strand (N-strand). The typical ATN (five with ATG, three with ATA, and two with ATT) are used as the start codon in most PCGs of these species, except for the *atp8*, *nad1*, and *cox1* genes, which use TTG as the start codon. *cox1*, *cox2*, and *atp6* sequences terminate with a single T, the terminal codon of *nad1* sequences is TAG, and the stop codon for the remaining genes was TAA.

**Figure 3.**
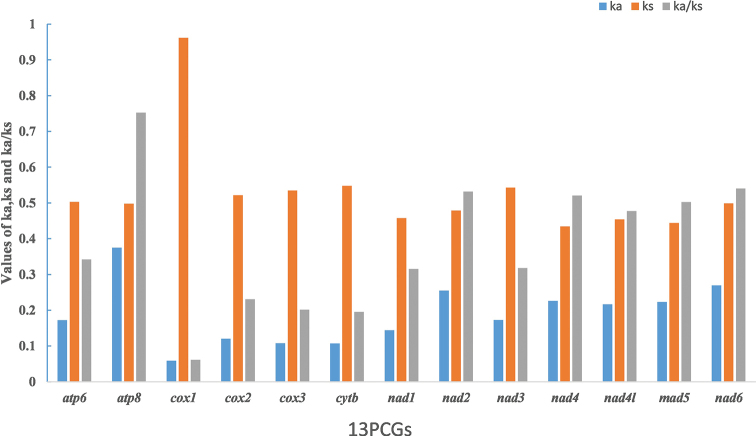
Evolutionary rates of 13 PCGs in Pentatomoidea. Rate of non-synonymous substitutions (Ka), rate of synonymous substitutions (Ks), and ratio of rate of non-synonymous substitutions to rate of synonymous substitutions (Ka/Ks) are calculated for each PCG.

In addition, we calculated non-synonymous substitutions (Ka), synonymous substitutions (Ks), and Ka/Ks ratios for the 13 PCGs of the Pentatomoidea (Fig. 3), and the evolutionary rates of the 13 PCGs are compared. The results clearly show that *atp8* evolved at the fastest rate (Ka/Ks = 0.75), *cox1* evolved at the slowest rate (Ka/Ks = 0.06), and the other genes evolved in the order of *nad6* > *nad2* > *nad4* > *nad5* > *nad4l* > *atp6* > *nad3* > *nad1* > *cox2* > *cox3* > *cytb*. Furthermore, all 13 PCGs have Ks values greater than Ka values and Ka/Ks ratios less than 1, indicating that these genes are affected by purifying selection.

### ﻿Transfer and ribosomal RNAs

**Figure 4.**
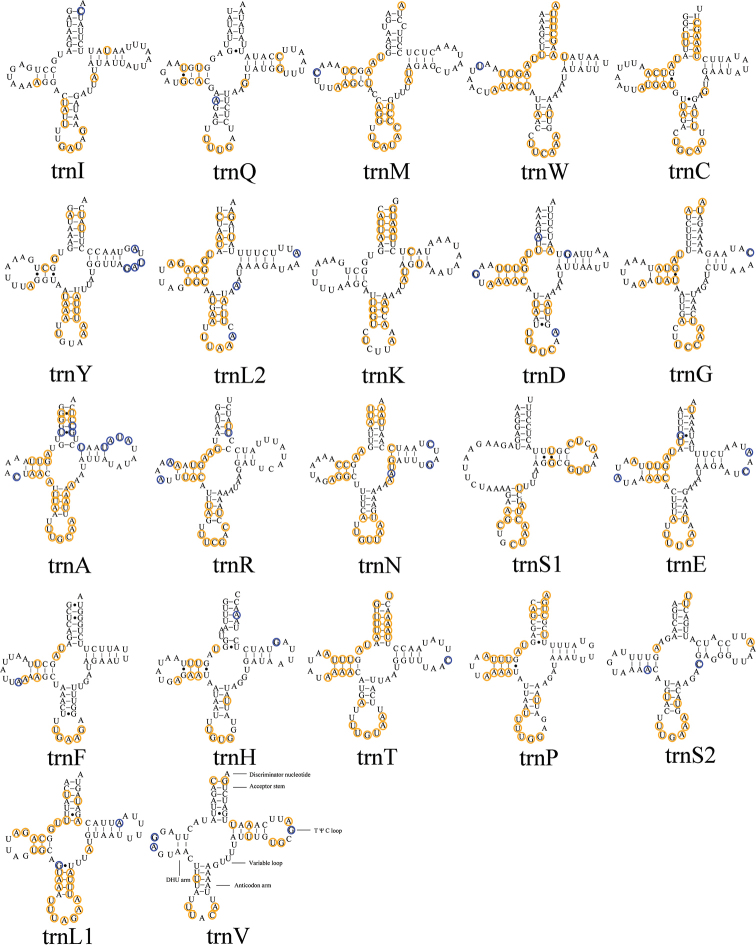
Predicted secondary structure of tRNA genes in *Aeschrocoristuberculatus*. Conserved sites in Pentatomoidea are marked orange. Nonconserved sites in *A.tuberculatus* and *A.ceylonicus* are marked blue.

The total lengths of the tRNAs of *A.tuberculatus* and *A.ceylonicus* are 1,503 bp and 1,502 bp, respectively. And the length of tRNA genes are from 64 bp to 72 bp. Fourteen genes (*trnA*, *trnE*, *trnD*, *trnG*, *trnK*, *trnI*, *trnL2*, *trnM*, *trnN*, *trnR*, *trnS1*, *trnS2*, *trnT*, and *trnW*) are located on the J-strand, and other eight genes on the N-strand. Only *trnS1* lacks a dihydrouridine (DHU) arm; the other tRNA genes all have the classic cloverleaf secondary structure. In addition to the typical base pairs (A-U and G-C), some wobble G-U pairs appear in these secondary structures, which can form stable chemical bonds between G and U (Fig. 4).

**Figure 5.**
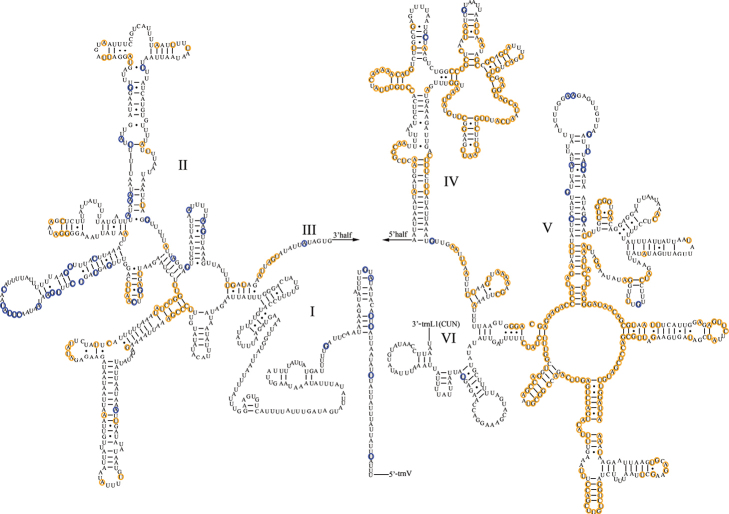
Predicted secondary structure of the *rrnL* in *Aeschrocoristuberculatus*. Conserved sites in Pentatomoidea are marked orange. Nonconserved sites in *A.tuberculatus* and *A.ceylonicus* are marked blue.

**Figure 6.**
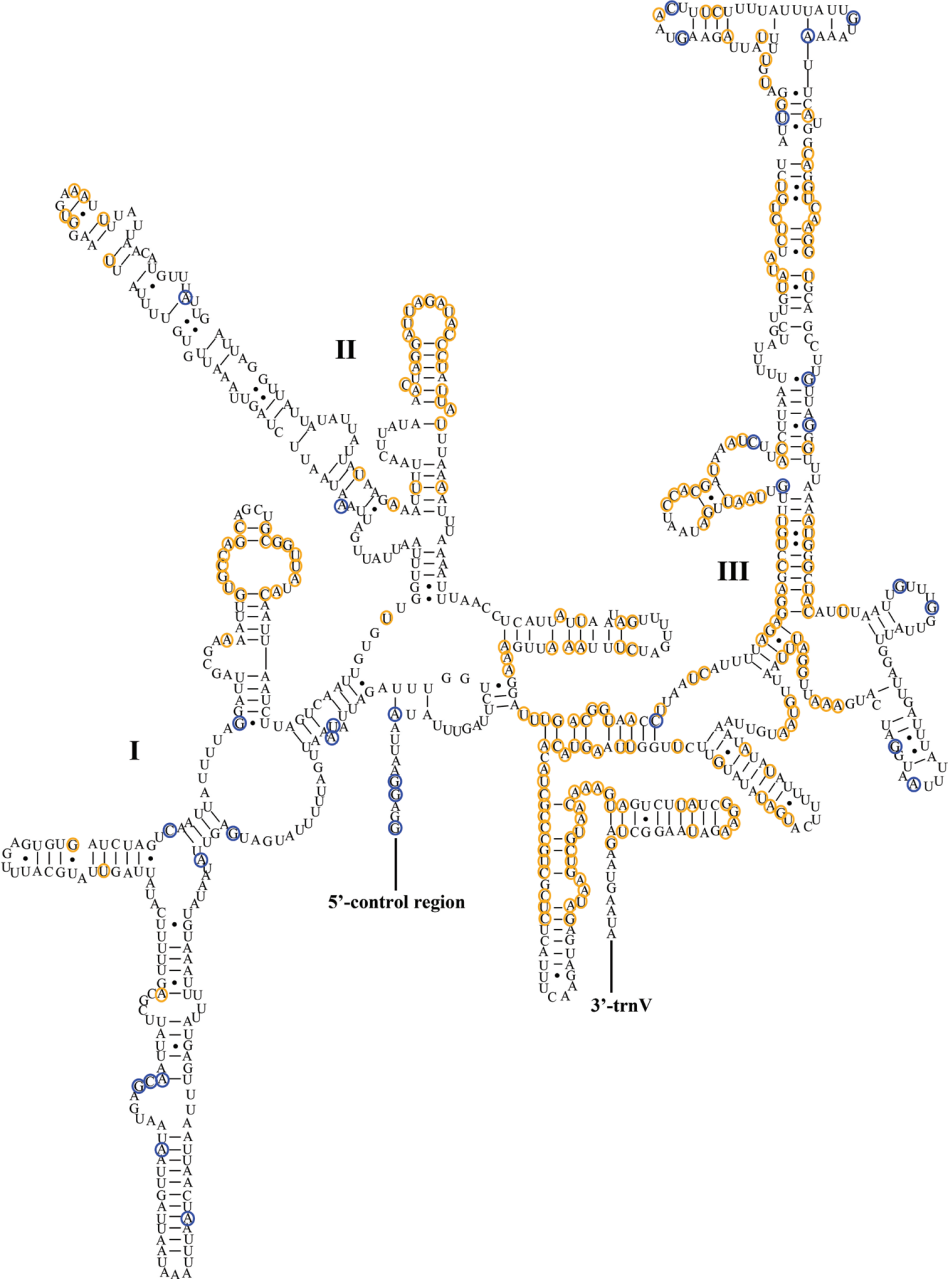
Predicted secondary structure of the *rrnS* in *Aeschrocoristuberculatus*. Conserved sites in Pentatomoidea are marked orange. Nonconserved sites in *A.tuberculatus* and *A.ceylonicus* are marked blue.

The *rrnL* and *rrnS* genes have the same situation in the two species. The *rrnL* gene is located between *trnL1* (CUN) and *trnV*, and the *rrnS* gene is located between *trnV* and the control region; they are encoded on the N-strand. The lengths of the two genes in *A.tuberculatus* are 1,309 bp (*rrnL*) and 809 bp (*rrnS*); the complete secondary structures are shown in Figs 5, 6. In *A.ceylonicus*, the two genes are 1,302 bp (*rrnL*) and 813 bp (*rrnS*) in length. The order of the base content of the rRNA genes is T (43.58%) > A (32.39%) > G (15.63%) > C (8.40%) and T (43.83%) > A (32.77%) > G (15.08%) > C (8.32%), respectively. The AT-skews are negative, and the GC-skews are positive.

### ﻿The control region

**Figure 7.**
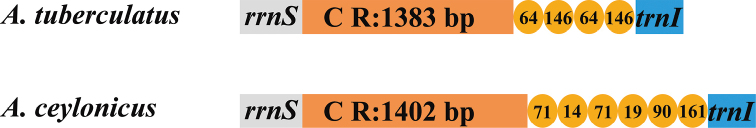
Organization of the control region in the mitochondrial genomes of *Aeschrocoristuberculatus* and *A.ceylonicus*. The tandem repeats are shown by yellow ovals with repeat length inside. CR indicates the length of the sequence of the control region.

The control is the main regulatory region for replication and transcription of the mitochondrial genome (Taanman 1999; Stewart and Beckenbach 2006; Cameron 2014). The variation in length of the control region is mainly caused by the lengths and numbers of repeating units. In conclusion, the sequence and structure of the mitochondrial control region is highly variable in Hemiptera (Moreno et al. 2010). The control region of *A.tuberculatus*, located between *rrnS* and *trnI* genes, is 1,383 bp in length, and the A + T content is 73.99%. The length of the control region of *A.ceylonicus*, at 1,402 bp, is similar to *A.tuberculatus*, and the A + T content is 77.35%. Moreover, both species have a variety of different tandem repeat units (Fig. 7).

### ﻿Tests of substitution saturation and heterogeneity

**Figure 8.**
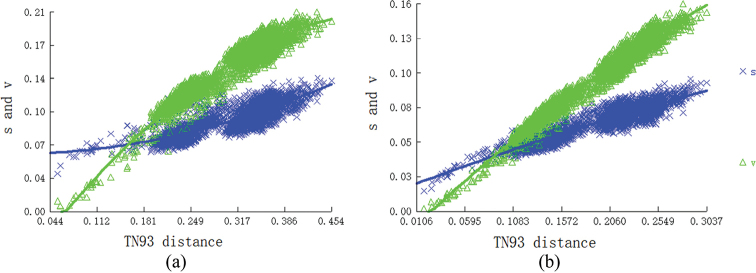
The substitution saturation analysis of two datasets **a** PCG123 **b** PCG12.

Before constructing the phylogenetic tree, we evaluated the substitution saturation of the PCG123 and PCG12 datasets. The results show that the Xia saturation index (Iss) is below the critical values for a symmetric (Iss.cSym) and asymmetric (Iss.cAsym) topology (Fig. 8). Meanwhile, the conversion rate and modified genetic distance both increase linearly, indicating that the nucleotide sequences of two datasets are not saturated.

**Figure 9.**
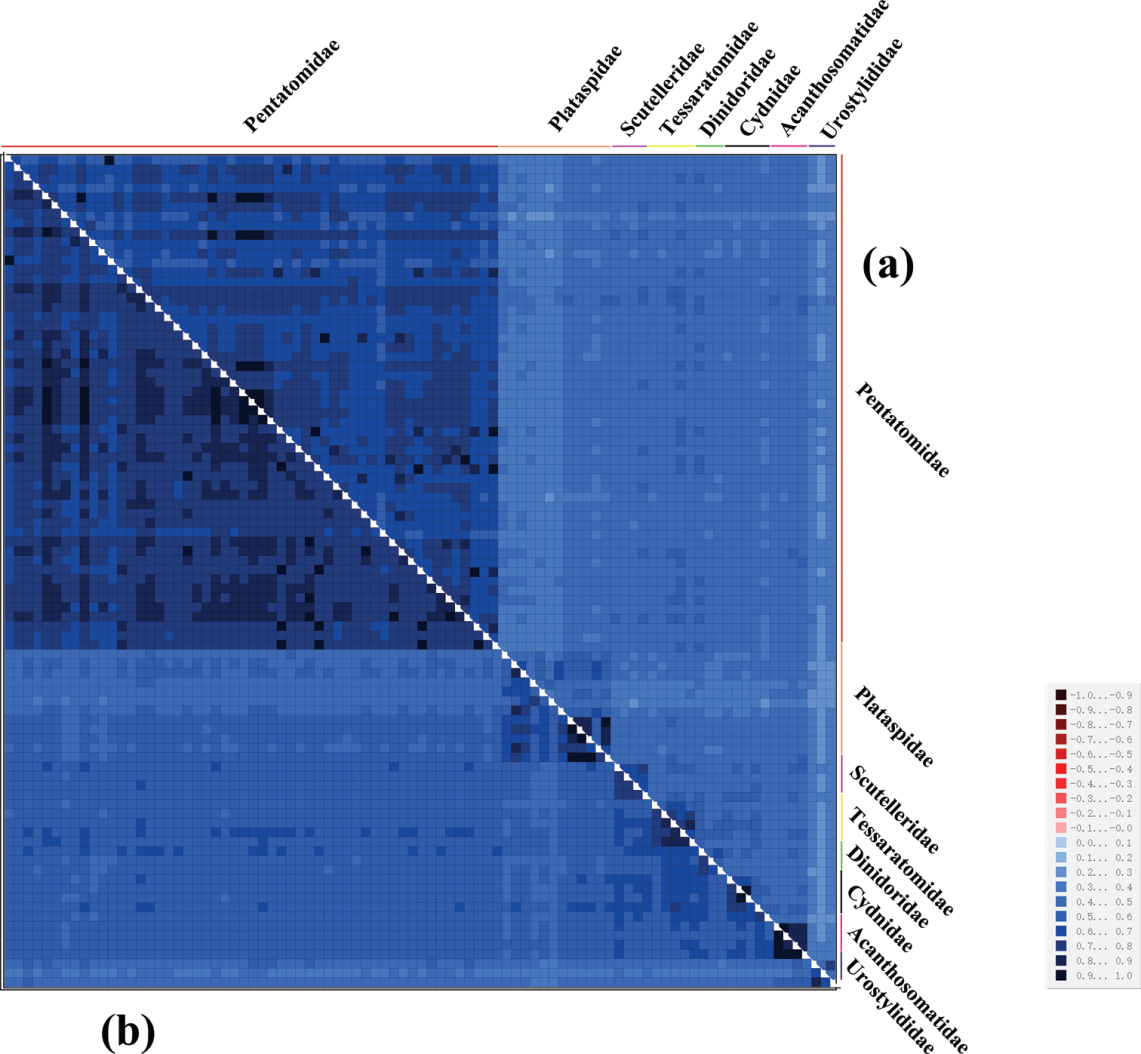
AliGROOVE analysis of 89 Pentatomoidea species **a** based on PCG123 **b** based on PCG12. The mean similarity score between sequences is represented by colored squares, based on AliGROOVE scores ranging from –1, which indicates a great difference in rates from the remainder of the data set (= heterogeneity, red color) to +1, which indicates rates that matched all other comparisons (blue color, as in this case).

Our analysis of the heterogeneity of the base composition in the two datasets show that the heterogeneity of PCG123 is higher than in PCG12, thus indicating a higher heterogeneity of the third site of the codon. The degree of heterogeneity between the two datasets is certainly consistent with the construction of a phylogenetic tree, which can be used for phylogenetic analysis (Fig. 9).

### ﻿Phylogenetic analyses

**Figure 10.**
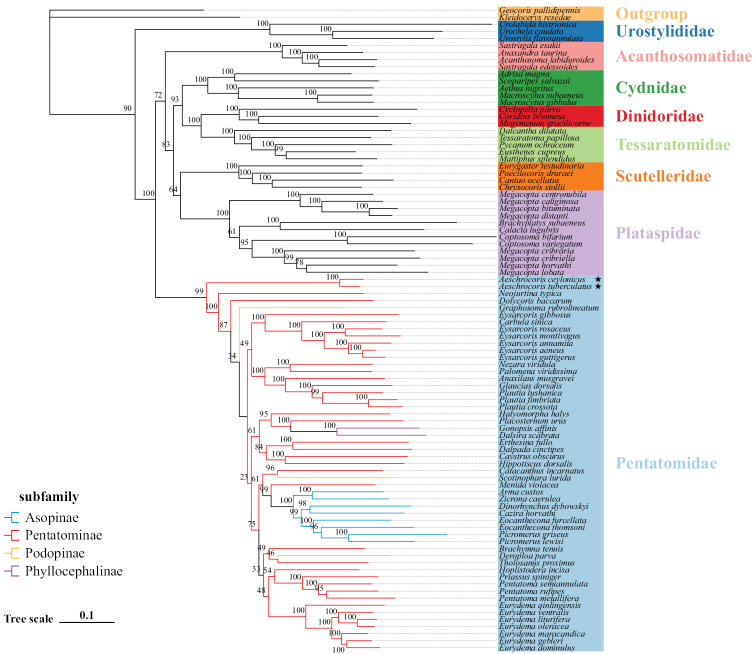
Phylogenetic tree using ML analyses based on PCG123. Numbers at the node are bootstrap values.

**Figure 11.**
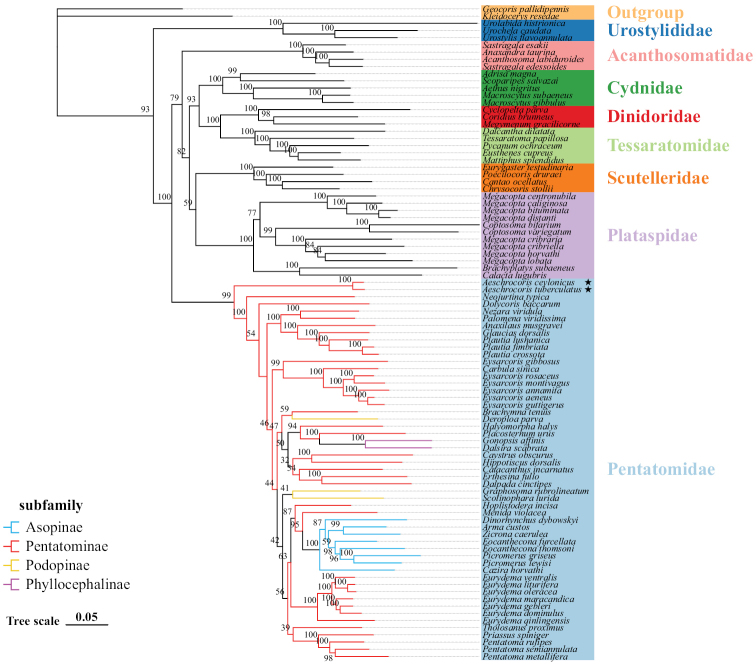
Phylogenetic tree using ML analyses based on PCG12. Numbers at the node are bootstrap values.

We constructed phylogenetic trees of Pentatomoidea based on the two datasets using the ML method (Figs 10, 11). The results show that the topological structure of the tree is reliable. The relationship is as follows: (Urostylididae + ((Acanthosomatidae + ((Cydnidae + (Dinidoridae + Tessaratomidae)) + (Scutelleridae + Plataspidae))) + Pentatomidae)). All analyses also show that *A.tuberculatus* and *A.ceylonicus* are the earliest diverging lineage within Pentatomidae and cluster as a sister group. The monophyly of Pentatominae and Podopinae is rejected, as both are scattered within the Pentatomidae clade. However, we recovered the monophyly of Asopinae and Phyllocephalinae with strong support values and high internal node support values. The two subfamilies are nested in one of the Pentatominae clades, so the subfamilies of Pentatomidae need further research.

## ﻿Discussion and conclusions

In this study, we sequenced and annotated the complete mitogenomes of *Aeschrocoristuberculatus* and *A.ceylonicus* using NGS technology and Geneious v. 11.0. Our analysis comparing of the mitochondrial genomes of the two species show that the gene arrangement is highly conserved, which is consistent with other published mitochondrial genomes of Hemiptera (Hua et al. 2008; Lee et al. 2009; Song et al. 2013). The lengths of the mitochondrial genomes of *A.tuberculatus* and *A.ceylonicus* are 16,134 bp and 16,142 bp, respectively. There were four overlapping regions in the mitochondrial genome of these two species. The positions of their overlapping regions re identical. One of the longest overlaps, located between *trnW* and *trnC*, is 8 bp in length, and the overlapping bases are AAGCTTTA, which is common in pentatomid species (Yuan et al. 2015; Zhao et al. 2019a). The other two pairs of genes, namely *atp8*/*atp6* and *nad4*/*nad4l*, overlap by 7 bp, and both overlapping bases are ATGATAA. Specifically, an overlap of 8 bp between *nad6* and *cytb* was also observed, and the overlapping bases are ATGAATAA. This is different from that found in previous studies on Pentatomidae. Between *trnS2* and *nad1*, the longest spacer region appeared in both, which is consistent with the findings of other studies (Hua et al. 2008; Zhao et al. 2019a). The difference of mitogenome size between *A.tuberculatus* and *A.ceylonicus* is due to the length difference of the noncoding region.

In most Pentatomidae mitochondrial genomes, only *cox1* has TTG as its start codon, and the remaining 12 PCGs use ATN as their start codon (Hua et al. 2008; Li et al. 2012). However, there is a difference between *A.tuberculatus* and *A.ceylonicus* in that nine PCGs have the same start codons ATN, and four PCGs (*nad1*, *cox1*, *atp8*, and *nad6*) use TTG as the start codon. Most PCGs use the TAA as the stop codon; nevertheless, in some insects, *nad1*, *cox2*, and some other genes use the single T or TAG as the stop codon (Liu et al. 2012; Song et al. 2013). In this study, our results show most PCGs stop with TAA, and three PCGs (*cox1*, *cox2*, and *atp6*) stop with a single T. However, one PCG (*nad1*) stops with TAG. In PCGs, *cox1* is commonly used for barcode analysis and genus or species identification due to its slow rate of evolution (Hebert et al. 2004).

The composition of the four bases in *A.tuberculatus* and *A.ceylonicus* is A>T>C>G. There is a clear AT preference in nucleotide composition. Most tRNAs have the typical cloverleaf secondary structure as observed in Hemiptera. However, the lack of a DHU arm in the *trnS1* is common in hemipteran mitogenomes (Wolstenholme 1992; Shi et al. 2012). *rrnL* and *rrnS* in *A.tuberculatus* and *A.ceylonicus* lie between *trnL1* (CUN) and *trnV*, and between *trnV* and the control region, respectively. In Pentatomoidea, *rrnS* contains 19.37% conserved sites and included three domains. The *rrnL* contains 26.81% conserved sites and six domains (domain III is absent), and the IV and V domains are relatively conservative.

Through the topological structure of the trees, the clade including Urostylididae is found to be the earliest clade lineage. It forms a sister group to the other families. The relationship of (Cydnidae + (Dinidoridae + Tessaratomidae)) was recovered in our phylogenetic results with high support; these results are consistent with previous studies (Grazia et al. 2008; Yuan et al. 2015; Wu et al. 2018; Zhao et al. 2018; Xu et al. 2021). Xu et al. (2021) used PCGRNA and PCG12RNA data sets to recover the sister-group relationship of (Plataspidae + Scutelleridae) and (Dinidoridae + Tessaratomidae), and we also obtained this result. Of course, there are still other conclusions to be made based on the phylogenetic studies of Pentatomoidea. Previously, two sister groups (Plataspidae + Pentatomidae) and (Cydnidae + Scutelleridae) were recovered (Zhao et al. 2018; Liu et al. 2019). Possible reasons include, for example, the number of samples, the selection of outliers, the selection of data sets, and the influence of branches. In addition, the saturation and heterogeneity of the third site of PCG has little effect on the topological structure of the trees. In the study of Hemiptera insects, retention of the third site of PCG does not reduce the reliability of the phylogenetic results (Fenn et al. 2008). Although in many studies, the results obtained from different data sets and inference methods show that there are some contradictions among the relationships among families, our results based on more species have higher reliability. This study also confirms that adding more mitochondrial genome sequences is the key to solve the phylogenetic relationships of Pentatomoidea at various different taxonomic levels.

We studied the genus *Aeschrocoris* at a molecular level for the first time and preliminarily identified its taxonomic position and evolution in phylogenetic relationships. This study not only discusses the relationships among families, but it also adds new molecular data for Pentatomidae. These results demonstrate that mitochondrial genomes can effectively reveal the phylogenetic relationships among differing taxonomic hierarchies. We should sequence more mitochondrial genes to provide greater evidence for exploring the phylogenetic relationships among taxa.
